# Bacterial conversion of a host weapon into a nutritional signal

**DOI:** 10.1016/j.jbc.2022.102600

**Published:** 2022-10-14

**Authors:** Miguel A. Valvano

**Affiliations:** Wellcome-Wolfson Institute for Experimental Medicine, Queen's University Belfast, Belfast, United Kingdom

**Keywords:** *Salmonella*, intracellular bacteria, oxidative stress, NADPH oxidase, DksA, DnaJ, nutritional immunity, oxygen tension, Type III secretion system, ROS, reactive oxygen species, SCV, *Salmonella*-containing vacuole

## Abstract

Bacteria engulfed by phagocytic cells must resist oxidation damage and adapt to cellular hypoxia, but the mechanisms involved in this process are not completely elucidated. Recent work by Kim *et al.* in the *Journal of Biological Chemistry* investigated how the intracellular pathogen *Salmonella enterica* activates gene expression required to counteract oxidative damage. The authors show that this bacterium utilizes host oxidative molecules to activate regulatory proteins that enhance the production of effector molecules, counteracting the host weapon NADPH oxidase and inducing a protective response.

Tissue inflammation and infection creates hypoxic microenvironments with very low oxygen tensions due to increased metabolic demands from the high density of inflowing inflammatory cells and microorganisms (1, 2, 3, 4, 5). This hypoxia can be compounded by limited tissue perfusion due to thrombosis, vasculature damage, compression of blood vessels by interstitial hypertension, and in some cases, either granuloma or abscess formation, all of which cause localized decrease in pH and reduction of the partial pressure of oxygen (6). Many bacterial pathogens can proliferate in microaerophilic and anaerobic environments, and infection hypoxia is thought to be both associated with bacterial invasion and dissemination and enhanced by the activation of immune cells through the production of reactive oxygen species (ROS), an activity that also consumes oxygen (2, 7). Unlike eukaryotic cells, bacterial pathogens can adapt to survive under low oxygen and further contribute to the hypoxic environment. Oxygen deprivation at the cellular level also limits ROS production by neutrophils and other phagocytic cells.

Hypoxia during infection has long been a phenomenon of interest (2, 5), especially at the organ and tissue levels. However, much less is known about the state of oxygen levels in the intracellular environment when cells become infected with pathogens that can survive intracellularly in membrane-bound compartments, making cellular infection-induced hypoxia an emerging field (6, 8). Adaptation of pathogens to hypoxia is controlled by regulatory proteins (6), which induce the expression of enzymes that assist bacterial adaptation to low oxygen and repress metabolic pathways such as the tricarboxylic acid cycle and the β-oxidation of fatty acids.

*Salmonella enterica* serovar Typhimurium is a model facultative intracellular pathogen that causes self-limiting gastroenteritis in humans and more severe infections in immunocompromised patients and patients with malaria (9). One of the critical pathogenicity mechanisms of *S. enterica* relies on the bacterium’s ability to survive and replicate in epithelial cells and macrophages within a modified bacteria-containing membrane compartment, known as the *Salmonella*-containing vacuole (SCV). *Salmonella* entry to nonphagocytic cells requires a specialized Type III secretory system (T3SS) encoded by the *Salmonella* pathogenicity island 1, which mediates the self-promoted uptake of the bacteria *via* modifications of the actin cytoskeleton. Upon phagocytosis by macrophages or self-promoted entry, *S. enterica* can remodel the SCV employing a T3SS encoded by the *Salmonella* pathogenicity island 2 (SPI-2). A complex array of regulators and regulatory signals controls the gene expression of the SPI-2 genes in response to nutritional cues arising from the intracellular SVC niche, such as acidic pH, osmolarity, and limitation in Mg^2+^ or inorganic phosphorous, all of which converge in the activation of the *ssrAB* locus encoded in SPI-2. The SsrB regulator protein, in turn, controls the expression of a T3SS also encoded in the same pathogenicity island, resulting in the assembly of a nanomachine capable of delivering protein effectors across the SCV membrane into the host cell cytosol.

The SsrB regulatory protein promotes gene transcription in two ways: by counteracting gene silencing by the histone-like nucleoid structuring protein, a major component of the folded chromosome in bacteria, and by concomitantly recruiting RNA polymerase to SPI-2 genes. This regulation is further fine-tuned by the combined actions on the kinetics of DNA–RNA polymerase open complexes of the nucleotide guanosine tetraphosphate (ppGpp), an alarmone (or second messenger molecule) that responds to environmental changes (10), and the α-helical protein DksA, both favoring SPI-2 gene transcription. Curiously, ppGpp and DksA are involved in the stringent response, a nutritional stress response that involves the activation of amino acid biosynthesis genes and the concomitant repression of translation. This suggests that nutritional stress in the SCV could be important for the adaptation of *Salmonella* in the intracellular niche (11). However, a key function of the SPI-2 T3SS is to antagonize the NADPH oxidase, an enzyme that produces potent antimicrobial ROS. The recent study by Kim *et al.* in JBC (12) describes that byproducts of the respiratory burst activate transcription by a mechanism that involves the DnaJ chaperone-dependent formation of DksA-RNA polymerase-SPI-2 gene ternary complexes.

The authors first demonstrated *in vitro* that significant *ssrB* gene transcription was stimulated by hydrogen peroxide (under low Mg^2+^ concentrations and in anaerobic conditions, as found in the SCV) and this upregulation required the DksA protein. However, the association of DksA with the RNA polymerase is not robust under oxidative stress due to the oxidation of key cysteine residues in the zinc finger domain of DksA. Because oxidized cysteine residues in DksA can be repaired by DnaJ and DnaK chaperones, the authors investigated whether one or both proteins was involved in stabilizing DksA function under oxidative conditions. They demonstrated that the DnaJ-K chaperone couple was required for DksA function and that DnaJ was the essential component involved. The *in vitro* experiments were replicated *in vivo*, demonstrating that the macrophage NAPH-oxidase activity triggered the activation of *ssrB*, and in turn, the expression *Salmonella* SPI-2 encoded genes, and this activation depends on DksA and DnaJ function (Fig. 1). It is likely that the host activation of the NADPH-oxidase also contributes to reduced oxygen tension in the SCV since the reaction consumes molecular oxygen (Fig. 1). Therefore, the anaerobic conditions in the SCV plus oxidative stress contribute to the expression of SPI-2 regulatory and effector genes in intracellular *Salmonella* through DksA and DnaJ functions. The link between the stringent response and antioxidant defense has been demonstrated for diverse bacterial pathogens (10, 13, 14, 15); however, while DnaJ and DksA are housekeeping proteins (13), the SPI-2 genes are acquired through horizontal gene transfer. The authors correctly propose that *Salmonella* has evolutionarily exploited inflammatory ROS production to induce a protective response mediated by SPI-2 genes and proteins against an important mechanism of host defenses based on the production of oxidative radicals (12).

**Figure 1:**
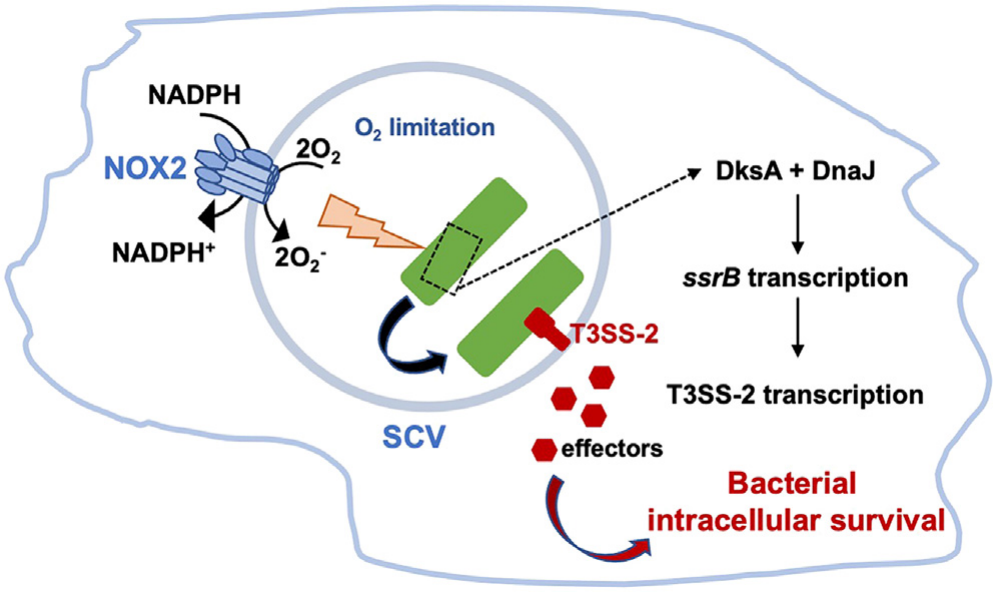
**Model of activation of the *Salmonella* T3SS-2 system upon DksA and DnaJ in response to host NADPH-dependent oxidative stress, which results in promoting bacterial intracellular survival.** NOX2, macrophage NADPH oxidase; SCV, *Salmonella*-containing vacuole.

The concept of cellular hypoxia upon infection is an emerging field (6). Several pathogens have evolved different mechanisms to overcome both reduced oxygen levels and the toxicity arising from ROS production. For example, one of these mechanisms involves rewiring the cell’s metabolism, as recently demonstrated for uropathogenic *Escherichia coli* (16), which requires aerobic respiration to establish infection in the bladder (*via* the bacterial quinol oxidase cytochrome *bd*, which supports intracellular bacterial replication and depletes oxygen from the urothelial cell cytosol). In contrast, the Gram-positive pathogen *Staphylococcus aureus* can directly interfere with the regulation of the host molecules hypoxia-inducible factor-1α and transforming growth factor β1 (TGF-β1) to promote bone infection (17). The findings of Kim *et al.* (12) in *S. enterica* reveal another mechanism for bacterial pathogens to harness the metabolic landscape of host cells and turn host cell responses into tools to establish themselves in relatively protected niches (Fig. 1).

## Conflict of interest

The author declares that he has no conflicts of interest with the contents of this article.
